# Optimal Resource Allocation Policies for Multi-User Backscatter Communication Systems

**DOI:** 10.3390/s16122016

**Published:** 2016-11-29

**Authors:** Bin Lyu, Zhen Yang, Guan Gui, Youhong Feng

**Affiliations:** 1 Key Laboratory of Ministry of Education in Broadband Wireless Communication and Senor Network Technology, Nanjing University of Posts and Telecommunications, Nanjing 210003, China; 13010511@njupt.edu.cn (B.L.); guiguan@njupt.edu.cn (G.G.); 2013010213@njupt.edu.cn (Y.F.); 2 College of Physics and Electronic Information Engineering, Anhui Normal University, Wuhu 241000, China

**Keywords:** backscatter communication, resource allocation, total goodput maximization

## Abstract

This paper considers a backscatter communication (BackCom) system including a reader and *N* tags, where each tag receives excitation signals transmitted by the reader and concurrently backscatters information to the reader in time-division-multiple-access (TDMA) mode. In this system, we aim to maximize the total system goodput by jointly optimizing reader transmission power, time allocation, and reflection ratio for the cases of passive and semi-passive tags. For each case, an optimization problem is formulated, which is non-convex and can be solved by being decomposed into at most *N* feasible sub-problems based on the priority of allocated reader transmission power. First, for the passive tags case, by solving the convex sub-problems sequentially and comparing their maximum total goodput, we derive the optimal resource allocation policy. Then, for the semi-passive tags case, we find a close-to-optimal solution, since each sub-problem can be reformulated as a biconvex problem, which is solved by a proposed block coordinate descent (BCD)-based optimization algorithm. Finally, simulation results demonstrate the superiority of the proposed resource allocation policies.

## 1. Introduction

With the emergence of the Internet-of-Things (IoT), the sustainable energy supply of electronic equipment has become an important issue. However, the lifetime of wireless devices with fixed sources (e.g., batteries) is limited, as periodically replacing or recharging batteries may be inconvenient or infeasible. Recently, radio frequency (RF) signals—which can charge devices by energy harvesting (EH) techniques—have become a potential energy source [1].

RF signals based on EH techniques have been extensively exploited in simultaneous wireless information and power transfer (SWIPT) systems [2,3] and wireless-powered communication networks (WPCNs) [4,5,6,7]. However, in either SWIPT systems or WPCNs, devices require oscillators for carrier signal generation and analog-to-digital converters (ADCs) for signal modulation, which causes high energy consumption. To solve this problem, backscatter communication (BackCom) techniques [8] that do not require oscillators and ADCs have received significant attention in recent years, and can be extensively used in wireless sensor networks [9,10,11,12]. BackCom systems normally include a transceiver, often known as a reader, and multiple nodes, known as tags. The reader transmits excitation signals to activate the tags in forward channels and receives backscattered signals from the tags in backward channels. Tags can be classified as three types: active, passive, and semi-passive. Active tags can radiate signals, while the latter two types communicate with readers based on reflection [8]. Throughout this paper, tags refer to passive or semi-passive tags without specific declaration. For an activated tag, there are typically two states—i.e., sleep and active states. In the sleep state, each tag’s antenna input impedance is matched, and all input energy will be harvested. In the active state, a portion of excitation signals is backscattered to the reader, and the remainder is harvested for circuit operation. However, only semi-passive tags can store energy during the sleep state, due to the availability of batteries.

In [13], ambient backscatter was introduced to enable ubiquitous communication where devices communicate by backscattering environment signals (e.g., TV broadcast signals). However, communication based on environmental power sources may not be stable. To fix this problem, fixed energy sources were introduced in [14]. In [15], a novel network architecture—i.e., a wirelessly powered BackCom network—was modeled using stochastic geometry, and the network coverage probability and transmission capacity were analyzed. In [16] and [17], the authors investigated the security problems of signal-input single-output (SISO) and multiple-input multiple output (MIMO) BackCom systems, respectively. To improve the transmission rate of the secondary user, the BackCom was introduced in cognitive radio networks [18]. To boost the deployment of BackCom systems, the reader collision problem was studied in [19].

The resource allocation problem is usually an important issue in wireless networks. In [20], the authors investigated the resource allocation schemes for two-tier networks, including spectrum-sharing femtocells and macrocells. In [21], the authors used cooperative Hash bargaining game theory to study the joint uplink subchannel and power allocation problem in cognitive small cells. Note that the time resource allocation of both the sleep and active states has an important effect on the performance of BackCom systems. However, this key factor was not investigated in the works [13,14,15,16,17]. Although this time allocation was considered in [18], the transmission rate was fixed such that the significant link reliability was not investigated. To account for the link reliability, the time allocation control for maximizing the system goodput with fixed reader transmission power in a single-user BackCom system including a semi-passive tag was studied in our previous work [22]. In practical deployment of a BackCom system, a reader usually serves multiple tags. Hence, this paper considers a practical multi-user BackCom system where each tag communicates with the reader in time-division-multiple-access (TDMA) mode. In this paper, both the passive and semi-passive tags are considered. Moreover, a dynamic reader transmission power allocation scheme is proposed to further improve the system performance. The key contributions of this paper are summarized as follows.

We consider a multi-user BackCom system including a reader and *N* tags for both cases of passive and semi-passive tags. To account for the link reliability of each case, an optimization problem is formulated to maximize the total goodput by jointly optimizing reader transmission power, time allocation, and reflection ratio.

For the case of passive tags, by exploiting the priority of allocated reader transmission power, the corresponding non-convex optimization problem is decomposed into *N* convex sub-problems. By solving the feasible sub-problems sequentially and comparing their maximum total goodput, we derive the optimal resource allocation policy. In this policy, a part of tags cannot be activated due to the low priorities. The “activate or not” decision is discussed.

For the case of semi-passive tags, the corresponding problems are also decomposed into *N* sub-problems. Each sub-problem is reformulated as a biconvex optimization problem, for which a local optimum point can be found by the proposed block coordinate descent (BCD) [23] based optimization algorithm. Finally, the close-to-optimal resource allocation policy is derived. Note that throughout this paper, “close-to-optimal” corresponds to “locally optimal”.

The simulation results are given to confirm the superiority of the proposed resource allocation policies. Moreover, the equal power allocation policy is given as the benchmark.

The rest of this paper is organized as follows. In Section 2, we introduce the system model. In Section 3 and Section 4, the problem formulation of the passive tags case is formulated, and the optimal solution for this problem is derived, respectively. In Section 5 and Section 6, for the case of semi-passive tags, the problem formulation and a close-to-optimal resource allocation policy are given, respectively. Simulation results are presented in Section 7. Finally, Section 8 concludes this paper.

## 2. System Model

**Figure 1:**
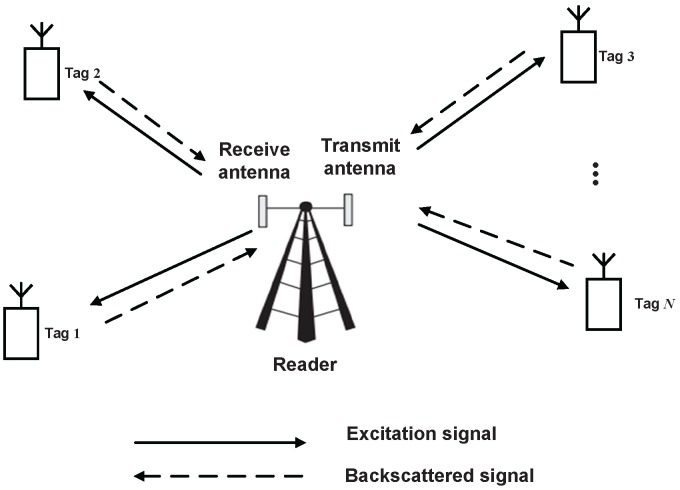
Multi-user backscatter communication system.

As shown in Figure 1, we consider a BackCom system consisting of one two-antenna reader and *N* single-antenna tags. BackCom is studied based on frame slots. The time duration of each frame is given as NT seconds. Each tag is equally allocated with a fixed time duration (given as *T* seconds), and can only work during its allocated duration. We assume that full-duplex mode at the reader is enabled by employing the perfect self-interference cancellation. During the duration of each tag, the reader transmits excitation signals to the tag and concurrently receives backscattered signals modulated by the tag.

### 2.1. Backscatter Channel

The forward and backward channels between the reader and *i*-th tag are modeled as free-space channels and reciprocal. The channel gains of the forward and backward channels—denoted by $h_{i}$ and $g_{i}$—remain constant over the frame slot. Moreover, the channel gain information is assumed to be perfectly known by the reader and tags. Therefore, the obtained total goodput of both cases of the passive and semi-passive tags using our proposed resource allocation policies are an upper bound of the total goodput with channel estimation errors in realistic scenarios.

### 2.2. Tag Configuration

Since batteries are only available for the semi-passive tags, the configurations of passive and semi-passive tags are quite different. Assuming the reader transmission power for the *i*-th tag as $P_{i}$, the receive power at this tag is given as $P_{i}^{\mathrm{rec}}=P_{i}h_{i}$. Considering the efficiency of both backscattered and harvested power, the signals are modulated by binary phase shift keying (BPSK) [24].

#### 2.2.1. Passive Tag

**Figure 2:**
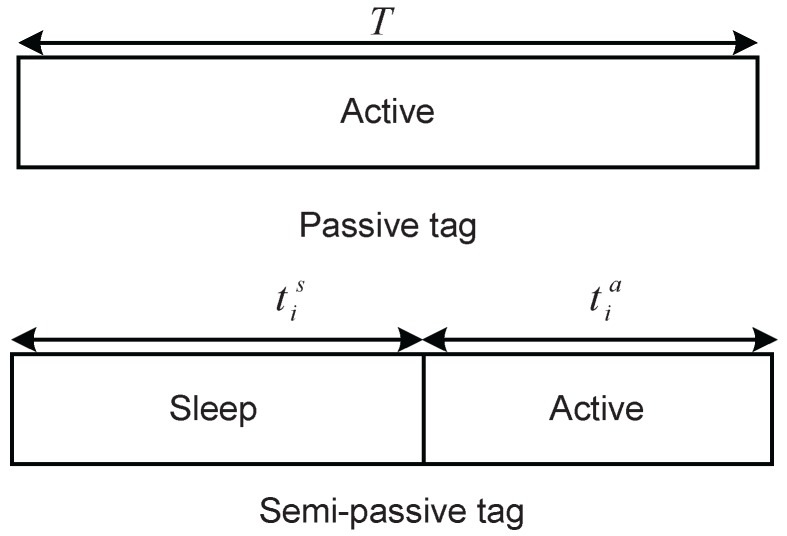
The working model of an activated tag.

For a passive tag, if the receive power exceeds a threshold, this tag is activated so that it can enter the active state directly without staying in the sleep state due to the absence of batteries. In the active state, the passive tag backscatters its signals using a portion of instantaneous excitation energy and harvests the remainder to support circuit power over the active time $t_{i}^{a}$, where $t_{i}^{a}=T$. The working model is shown in Figure 2. Without loss of generality, assume that the circuit power and energy harvesting efficiency of all passive tags are same, denoted by $P^{c}$ and *η*, respectively. Since BPSK is employed, define $n_{i}$ as the reflection ratio for signal backscattering of the *i*-th passive tag, following the definition in [24]. Hence, the backscattered power of the *i*-th passive tag (denoted by $P_{i}^{b}$) is given as $P_{i}^{b}=n_{i}P_{i}^{\mathrm{rec}}$. The harvested energy to cover the circuit energy consumption is given as $E_{i}^{h}=\eta \left(1-n_{i}\right)P_{i}^{\mathrm{rec}}T$. If the receive power is lower than the threshold, the passive tag cannot be activated, so no signals will be backscattered.

#### 2.2.2. Semi-Passive Tag

For a semi-passive tag, we assume that the battery does not have energy at the beginning. First, the *i*-th semi-passive tag enters the sleep state to harvest energy while consuming negligible circuit power over sleep time $t_{i}^{s}$ [8]. For symbol simplification, the definitions of the semi-passive tags case are the same ones as those of the passive tags case. The stored energy of the *i*-th semi-passive tag in the sleep state (denoted by $E_{i}^{s}$) is given as $E_{i}^{s}=\eta P_{i}^{\mathrm{rec}}t_{i}^{s}$. Then, the *i*-th semi-passive tag operates in the active state over the remaining time $t_{i}^{a}$, satisfying $t_{i}^{a}=T-t_{i}^{s}$, which is shown in Figure 2. The other definitions of the *i*-th semi-passive tag during the active state can refer to those of the passive tags case.

### 2.3. Performance Metric

Define the bit error rate (BER) corresponding to the *i*-th tag at the reader as $p_{i}^{e}$. According to [25], $p_{i}^{e}$ is given as

$$p_{i}^{e}=\frac{1}{2}\mathrm{erfc}\left(\sqrt{\frac{P_{i}^{b}g_{i}}{\sigma^{2}}}\right)=\frac{1}{2}\mathrm{erfc}\left(\sqrt{a_{i}n_{i}P_{i}}\right),$$

where $a_{i}=\frac{h_{i}g_{i}}{\sigma^{2}}$ and $\sigma^{2}$ is the noise power at the reader. To make the system more practical, the quality of service (QoS) is considered. Denote the maximum BER that the system can tolerate as *ε* (i.e., $p_{i}^{e}\leq \varepsilon$).

The successful transmission bits of each symbol in the active state—named as *goodput*—can be given as

$$\begin{array}{cc} G_{i} & =G_{i}\left(P_{i},t_{i}^{a},n_{i}\right)=t_{i}^{a}\left(1-p_{i}^{e}\right)=t_{i}^{a}\left[\frac{1}{2}+\frac{1}{2}\mathrm{erf}\left(\sqrt{a_{i}n_{i}P_{i}}\right)\right], \end{array}$$

where function erfc(x)=1−erf(x). The performance metric, *total goodput*, is given as $\sum_{i=1}^{N}G_{i}$.

## 3. Problem Formulation of Passive Tags

In this section, the total goodput maximization problem of a BackCom system with *N* passive tags is considered. As stated before, the *i*-th passive tag can be activated, only when the corresponding reader transmission power is not smaller than a threshold. The threshold is given as

$$P_{i}^{\mathrm{th}}=\frac{P^{c}}{\eta h_{i}}+\frac{{\left[{\mathrm{erfc}}^{-1}\left(2\varepsilon \right)\right]}^{2}}{a_{i}},\;\forall i.$$

To avoid the waste of power, if the reader transmission power for the *i*-th passive tag is smaller than its threshold, let $P_{i}=0$ to allocate more power to other tags with better channel conditions. Therefore, the reader transmission power for the *i*-th tag satisfies

(1) $$\begin{array}{cc} P_{i} & \left\{\begin{array}{c} >0,\;P_{i}\geq P_{i}^{\mathrm{th}}\\ =0,\;P_{i}<P_{i}^{\mathrm{th}}. \end{array}\right. \end{array}$$

Based on the above analysis, the goodput formulation is rewritten as

(2) $$\begin{array}{cc} G_{i}\left(P_{i},n_{i}\right) & =\left\{\begin{array}{cc} T\left[\frac{1}{2}+\frac{1}{2}\mathrm{erf}\left(\sqrt{a_{i}n_{i}P_{i}}\right)\right],\; & P_{i}\geq P_{i}^{\mathrm{th}}\\ 0,\; & P_{i}<P_{i}^{\mathrm{th}}. \end{array}\right. \end{array}$$

Once the *i*-th passive tag is activated, the circuit energy constraint $P^{c}T\leq E_{i}^{h}$ should be satisfied, from which $n_{i}$ is rewritten as $n_{i}\leq n_{i}^{\max}=\frac{P_{i}h_{i}\eta -P^{c}}{P_{i}h_{i}\eta }$. Considering the QoS constraint, the minimum reflection ratio for the *i*-th tag is given as $n_{i}^{\min}=\frac{{\left[{\mathrm{erfc}}^{-1}\left(2\varepsilon \right)\right]}^{2}}{a_{i}P_{i}}$. Therefore, the reflection ratio for the *i*-th tag satisfies $n_{i}^{\min}\leq n_{i}\leq n_{i}^{\max}$. The reader transmission power for each tag is also constrained by the average and peak power, which are denoted by $P^{\mathrm{ave}}$ and $P^{\max}$, respectively. The constraints are given as $\frac{1}{N}\sum_{i=1}^{N}P_{i}\leq P^{\mathrm{ave}}$ and $P_{i}\leq P^{\max},\forall i$, respectively. Therefore, the optimization problem is formulated as follows to maximize the total goodput.

$$\begin{array}{ccc} \left(P1\right)\;\;\max_{P,n}\;\;\; & \sum_{i=1}^{N}G_{i}\left(P_{i},n_{i}\right) & \\ s.t.\; & P_{i}=P_{i}1\left(P_{i}\geq P_{i}^{\mathrm{th}}\right), & \;\forall i,\\ & P_{i}\leq P^{\max}, & \;\forall i,\\ & n_{i}^{\min}1\left(P_{i}\geq P_{i}^{\mathrm{th}}\right)\leq n_{i}\leq n_{i}^{\max}1\left(P_{i}\geq P_{i}^{\mathrm{th}}\right), & \;\forall i,\\ & \frac{1}{N}\sum_{i=1}^{N}P_{i}\leq P^{\mathrm{ave}}, & \end{array}$$

where $P=[P_{1},\ldots ,P_{N}]$ and $n=[n_{1},\cdots ,n_{N}]$, 1(·) is the indicator function, having the Value 1 if the condition $P_{i}\geq P_{i}^{\mathrm{th}}$ is satisfied, or the value 0 if the condition is not satisfied.

Problem P1 is a non-convex problem because the constraints include the indicator function. Hence, it is difficult to find the optimal solution by solving Problem P1 directly. In the following section, the optimal solution for Problem P1 is investigated by exploiting the priority of allocated reader transmission power.

## 4. Optimal Resource Allocation of Passive Tags

In this section, the optimal solution for Problem P1 is derived. First, Problem P1 is decomposed into *N* convex sub-problems. Then, the optimal solutions for these feasible sub-problems are derived by convex optimization techniques. Last, the solution for the sub-problem with the maximum total goodput also solves Problem P1.

$G_{i}\left(P_{i},n_{i}\right)$ is an increasing function with respect to $P_{i}$. To maximize the total goodput, these tags with better channel conditions will be allocated power (or be activated) with priority. Hence, there are at most *N* permutations for activated passive tags. Without loss of generality, assuming the channel gains of *N* passive tags satisfy $h_{1}\geq h_{2}\geq \ldots \geq h_{N}$, these *N* permutations for the indices of activated passive tags are given as [1,…,N], [1,…,N−1], …, [1]. Based on the above permutations, Problem P1 can be decomposed into *N* sub-problems. That is to say, each permutation corresponds to a sub-problem. For the *i*-th sub-problem, the number of activated passive tags is *M*, where M=N−i+1. $G_{i}\left(P_{i},n_{i}\right)$ is also an increasing function with respect to $n_{i}$, so that $n_{i}$ can keep as $n_{i}=n_{i}^{\max}$ over the whole duration in order to maximize the power of backscattered signals. The *i*-th sub-problem is given by

$$\begin{array}{ccc} \left(P2\right)\;\;\max_{\hat{P}}\;\;\; & \sum_{m=1}^{M}{\hat{G}}_{m}\left(P_{m}\right) & \\ s.t.\; & P_{m}^{\mathrm{th}}\leq P_{m},\;P_{m}\leq P^{\max},\; & \forall m,\\ & \sum_{m=1}^{M}P_{m}\leq NP^{\mathrm{ave}}, & \end{array}$$

where ${\hat{G}}_{m}\left(P_{m}\right)=T\left[\frac{1}{2}+\frac{1}{2}\mathrm{erf}\left(\sqrt{a_{m}P_{m}-b_{m}}\right)\right]$, $b_{m}=\frac{P^{c}g_{m}}{\sigma^{2}\eta }$, and $\hat{P}=\left[P_{1},\ldots ,P_{M}\right]$. It can prove that ${\hat{G}}_{m}\left(P_{m}\right)$ is a concave function by deriving its second derivative with respect to $P_{m}$. It follows that $\sum_{m=1}^{M}{\hat{G}}_{m}\left(P_{m}\right)$ is also a concave function, since it is the summation of ${\hat{G}}_{m}\left(P_{m}\right)$. Hence, Problem P2 is a convex problem [26]. Before solving the *i*-th sub-problem, we first check its feasibility. The feasibility of Problem P2 is given as $P_{m}^{\mathrm{th}}\leq P^{\max},\forall m$ and $\sum_{m=1}^{M}P_{m}^{\mathrm{th}}\leq NP^{\mathrm{ave}}$. If Problem P2 is feasible, the optimal solution for Problem P2 can be derived by considering two sub-cases. First, check whether $NP^{\mathrm{ave}}\geq MP^{\max}$. If so, the optimal solution for Problem P2 can be easily derived below.

**Theorem** **1.** *The optimal power allocation of Problem P2 under the condition* $NP^{ave}\geq MP^{max}$ *is given by*

$$P_{m}^{*}=P^{max},\;\forall m.$$

In order to derive the optimal solution for Problem P1, all feasible sub-problems need to be solved sequentially. To reduce the complexity, a useful conclusion is given in the following lemma.

**Lemma** **1.** *If* $NP^{ave}\geq MP^{max}$ *for the i-th sub-problem, then the optimal solutions for* i+1*-th, ..., N-th sub-problem are not the optimal solution for Problem P1.*

**Proof.**  See Appendix A. ☐

Based on Lemma 1, it is not necessary to solve the i+1-th, ..., *N*-th sub-problem if $NP^{\mathrm{ave}}\geq MP^{\max}$, since the optimal solution for Problem P1 can be found from the first *i* sub-problems.

If $NP^{\mathrm{ave}}<MP^{\max}$, Problem P2 is solved by Lagrange method. The optimal power allocation is given in the following theorem.

**Theorem** **2.** *If* $NP^{ave}<MP^{max}$*, then the optimal power allocation for Problem P2 is given as follows*

$$P_{m}^{*}=\min\left\{\max\left\{P_{m}^{th},\frac{\nu_{m}^{*}+b_{m}}{a_{m}}\right\},P^{max}\right\},\;\forall m,$$

*where* $\nu_{m}^{*}$ *is the unique solution for the equation* $G\left(\nu_{m},c_{m}\right)-\lambda^{*}=0$ *with respect to* $\nu_{m}$, $G\left(\nu_{m},c_{m}\right)=\frac{1}{2\sqrt{\pi }}c_{m}e^{-\nu_{m}}\nu_{m}^{-\frac{1}{2}}$, $c_{m}=a_{m}T$, $\lambda^{*}$ *is the optimal Lagrange multiplier, and* $\sum_{m=1}^{M}P_{m}^{*}=NP^{ave}$*.*

**Proof.**  See Appendix B. ☐

The optimal Lagrange multiplier $\lambda^{*}$ can be obtained by the bisection method. Based on above discussions, the optimal power allocation policy can be derived using Algorithm 1. The asymptotic complexity of Algorithm 1 is analyzed as follows. The main complexity of Problem P2 comes from deriving Theorem 2. Suppose Δ iterations are needed for the bisection method used in Theorem 2 to guarantee the converge. The complexity of deriving Theorem 2 is given as O(ΔM). We assume that $NP^{\mathrm{ave}}\geq \left(N-i+1\right)P^{\max}$ if and only if i>K (K≤N) and consider the worst-case; i.e., the first *K* sub-problems are all feasible, the total complexity of Algorithm 1 is thus O(KΔM).

**Algorithm 1** Algorithm for Problem P1
**Step 1**: Decompose Problem P1 into *N* sub-problems given as Problem P2, and let i=1;**Step 2**: If i≤N, check the feasibility of the *i*-th sub-problem. Then, if the *i*-th sub-problem is feasible, turn to Step 3; else, let i=i+1 and turn to Step 2. If i>N, turn to Step 4.**Step 3**: If $NP^{\mathrm{ave}}\geq MP^{\max}$, the optimal power allocation of the *i*-th sub-problem is given in Theorem 1 and turn to Step 4; If not, the optimal power allocation of *i*-th sub-problem is given in Theorem 2, let i=i+1, and turn to Step 2.**Step 4**: Compare the total goodput of all feasible sub-problems. The solution corresponding to the maximum total goodput also solves Problem P1.

**Remark 1** (Activate or Not)**.** *It can be derived that the decision on whether a tag can be activated is determined by the allocated reader transmission power (or the receive power at the tag). If the allocated power* $P_{i}$ *is not smaller than the threshold* $P_{i}^{th}$*, the i-th tag is activated; otherwise, this tag stays in the non-activated state. From Algorithm 1, if the i-th sub-problem corresponds to the maximum total goodput, only the first* N−i+1 *tags can be activated.*

Generally, for a BackCom system with passive tags, the tags corresponding to worse channel conditions cannot be activated because the average and peak of reader transmission power are limited. To make the system work in lower reader transmission power, a BackCom system with semi-passive tags is considered in the following sections.

## 5. Problem Formulation of Semi-Passive Tags

In this section, the total goodput maximization problem of a BackCom system with *N* semi-passive tags is studied. Since each semi-passive tag has a battery, it can first enter the sleep state to store energy and then operate in the active state. Therefore, the activation threshold for the *i*-th semi-passive tag can be reduced compared to the case of passive tags. This threshold is given as follows:

$$P_{i}^{\mathrm{th}}=\frac{{\left[{\mathrm{erfc}}^{-1}\left(2\varepsilon \right)\right]}^{2}}{a_{i}},\;\forall i.$$

Following the case of passive tags, Equation (1) is also suitable here, while $t_{i}^{a}$ needs to further optimized. Hence, Equation (2) is rewritten as

$$\begin{array}{cc} G_{i}\left(P_{i},t_{i}^{a},n_{i}\right) & =\left\{\begin{array}{cc} t_{i}^{a}\left[\frac{1}{2}+\frac{1}{2}\mathrm{erf}(\sqrt{a_{i}n_{i}P_{i}})\right],\; & P_{i}\geq P_{i}^{\mathrm{th}}\\ 0,\; & P_{i}<P_{i}^{\mathrm{th}}. \end{array}\right. \end{array}$$

If the *i*-th semi-passive tag can be activated, the reflection ratio satisfies $n_{i}^{\min}\leq n_{i}\leq 1$. To power up the *i*-th semi-passive tag, the energy harvested during both the sleep and active states should be sufficient to cover the circuit energy consumption, resulting in the following circuit power constraint:

$$\begin{array}{c} P^{c}t_{i}^{a}\leq \eta P_{i}h_{i}\left(T-t_{i}^{a}\right)+\eta \left(1-n_{i}\right)P_{i}h_{i}t_{i}^{a},\;\forall i. \end{array}$$

Based on the above discussions, the optimization problem of maximizing the total goodput is given as follows

$$\begin{array}{ccc} \left(P3\right)\;\;\max_{P,t^{a},n}\;\;\; & \sum_{i=1}^{N}G_{i}\left(P_{i},t_{i}^{a},n_{i}\right) & \\ s.t.\; & P_{i}=P_{i}1\left(P_{i}\geq P_{i}^{\mathrm{th}}\right), & \;\forall i,\\ & P_{i}\leq P^{\max},\;0\leq t_{i}^{a}\leq T, & \;\forall i,\\ & n_{i}^{\min}1\left(P_{i}\geq P_{i}^{\mathrm{th}}\right)\leq n_{i}\leq 1\left(P_{i}\geq P_{i}^{\mathrm{th}}\right), & \;\forall i,\\ & P^{c}t_{i}^{a}\leq \eta P_{i}h_{i}\left(T-t_{i}^{a}\right)+\eta \left(1-n_{i}\right)P_{i}h_{i}t_{i}^{a}, & \;\forall i\\ & \frac{1}{N}\sum_{i=1}^{N}P_{i}\leq P^{\mathrm{ave}}, & \end{array}$$

where $t^{a}=\left[t_{1}^{a},\cdots ,t_{N}^{a}\right]$.

Problem P3 is also a non-convex problem. Different from the case of passive tags, Problem P3 is more complicated. In the following section, both the priority of the allocated reader transmission power and the biconvex property are used to solve Problem P3.

## 6. The Optimal Resource Allocation of Semi-Passive Tags

Like the case of passive tags, the allocated reader transmission power controls the decision to “activate or not”. Hence, there are also at most *N* permutations for activated semi-passive tags. Based on the analysis in Section 4, Problem P3 is also decomposed into *N* sub-problems. The formulation of the *i*-th sub-problem is given by

$$\begin{array}{ccc} \left(P4\right)\;\;\max_{\hat{P},{\hat{t}}^{a},\hat{n}}\;\;\; & \sum_{m=1}^{M}t_{m}^{a}\left[\frac{1}{2}+\frac{1}{2}\mathrm{erf}(\sqrt{a_{m}P_{m}n_{m}})\right] & \\ s.t.\; & P_{m}^{\mathrm{th}}\leq P_{m},\;P_{m}\leq P^{\max}, & \forall m,\\ & 0\leq t_{m}^{a}\leq T,\;n_{m}^{\min}\leq n_{m}\leq 1, & \forall m,\\ & P^{c}t_{m}^{a}\leq \eta P_{m}h_{m}\left(T-t_{m}^{a}\right)+\eta \left(1-n_{m}\right)P_{m}h_{m}t_{m}^{a}, & \forall m,\\ & \sum_{m=1}^{M}P_{m}\leq NP^{\mathrm{ave}}, & \end{array}$$

where ${\hat{t}}^{a}=\left[t_{1}^{a},\ldots ,t_{M}^{a}\right]$ and $\hat{n}=\left[n_{1},\ldots ,n_{M}\right]$. The feasible condition of Problem P4 is also given as $P_{m}^{\mathrm{th}}\leq P^{\max},\forall m$ and $\sum_{m=1}^{M}P_{m}^{\mathrm{th}}\leq NP^{\mathrm{ave}}$. Assume Problem P4 is feasible. Before solving Problem P4, a useful structure for the optimal solution is given in the following lemma.

**Lemma** **2.** *The optimal* $P_{m}^{*}$, $t_{m}^{a*}$, and $n_{m}^{*}$ *for solving Problem P4 satisfy:*

$$P^{c}t_{m}^{a*}=\eta P_{m}^{*}h_{m}\left(T-t_{m}^{a*}\right)+\eta \left(1-n_{m}^{*}\right)P_{m}^{*}h_{m}t_{m}^{a*},\;\forall m.$$

**Proof.**  See Appendix C. ☐

To solve Problem P4, two sub-cases mentioned in Section 4 are considered. If $NP^{\mathrm{ave}}\geq MP^{\max}$, let $P_{m}^{*}=P^{\max},\forall m$. Lemma 2 indicates that extending the active time by full use of harvested power leads to the maximum total goodput. With Lemma 2 and $P_{m}^{*}=P^{\max},\forall m$, $n_{m}$ is rewritten as $n_{m}=\frac{T}{t_{m}^{a}}-\frac{P^{c}}{\eta P^{\max}h_{m}}$. Combining with the constraints $n_{m}^{\min}\leq n_{m}\leq 1$ and $0\leq t_{m}^{a}\leq T$ leads to the new constraint $\frac{\eta P^{\max}h_{m}T}{P^{c}+\eta P^{\max}h_{m}}\leq t_{m}^{a}\leq \hat{T}$, where ${\hat{T}}_{m}=\min\left\{\frac{\eta P^{\max}h_{m}T}{P^{c}+\eta n_{m}^{\min}P^{\max}h_{m}},T\right\}$. Based on the above analysis, Problem P4 can be reduced to Problem P5 as follows.

$$\begin{array}{cc} \left(P5\right)\;\;\max_{{\hat{t}}^{a}}\;\;\; & \sum_{m=1}^{M}t_{m}^{a}\left[\frac{1}{2}+\frac{1}{2}\mathrm{erf}\left(\sqrt{\frac{\gamma_{m}}{t_{m}^{a}}-b_{m}}\right)\right]\\ s.t.\; & \frac{\eta P^{\max}h_{m}T}{P^{c}+\eta P^{\max}h_{m}}\leq t_{m}^{a}\leq {\hat{T}}_{m},\;\forall m, \end{array}$$

where $\gamma_{m}=c_{m}P^{\max}$. It is easy to prove that Problem P5 is a convex problem. Exploiting the Lagrange method and KKT conditions, the optimal active time and reflection ratio with $P_{m}^{*}=P^{\max},\forall m$ are given in the following theorem.

**Theorem** **3.** *Given the reader transmission power of each activated semi-passive tag as* $P^{max}$*, the optimal* $t_{m}^{a*}$ *and* $n_{m}^{*}$ *have the following structure.*

$$t_{m}^{a*}=\min\left\{\max\left\{\frac{\eta P^{max}h_{m}T}{P^{c}+\eta P^{max}h_{m}},\frac{\gamma_{m}}{z_{m}^{*}+b_{m}}\right\},{\hat{T}}_{m}\right\},$$

$$n_{m}^{*}=\frac{T}{t_{m}^{a*}}-\frac{P^{c}}{\eta P^{max}h_{m}},$$

*where* $z_{m}^{*}$ *is the solution for* $f(z_{m},b_{m})=0$ *with respect to* $z_{m}$*,* $f\left(z_{m},b_{m}\right)=\frac{1}{2}+\frac{1}{2}\mathrm{erf}\left(\sqrt{z_{m}}\right)-\frac{1}{2\sqrt{\pi }}\left(z_{m}+b_{m}\right)z_{m}^{-\frac{1}{2}}e^{-z_{m}}$*.*

**Proof.**  Please refer to [22]. ☐

Theorem 3 reveals that the optimal control policy of an activated tag has a threshold-based structure. The thresholds are with respect to the reader transmission power. Normally, an activated semi-passive tag has two working strategies; i.e., *directly active* or *sleep-then-active*. The decision on whether the activated semi-passive tag first enters the sleep state is characterized in [22].

If $NP^{\mathrm{ave}}<MP^{\max}$, the method used for Problem P5 cannot solve Problem P4, due to the coupling of $P_{m}$, $t_{m}^{a}$ and $n_{m}$ in the objective function and constraints. However, Problem P4 can be reformulated as Problem P6 based on Lemma 2,

$$\begin{array}{ccc} \left(P6\right)\;\;\max_{\hat{P},{\hat{t}}^{a}}\;\;\; & \sum_{m=1}^{M}t_{m}^{a}\left[\frac{1}{2}+\frac{1}{2}\mathrm{erf}\left(\sqrt{\frac{c_{m}P_{m}}{t_{m}^{a}}-b_{m}}\right)\right] & \\ s.t.\; & P_{m}^{\mathrm{th}}\leq P_{m}\leq P^{\max},\; & \forall m,\\ & 0\leq t_{m}^{a}\leq T,\; & \forall m,\\ & \frac{\eta P_{m}h_{m}T}{P^{c}+\eta P_{m}h_{m}}\leq t_{m}^{a}\leq \frac{\eta P_{m}h_{m}T}{P^{c}+\delta_{m}},\; & \forall m,\\ & \sum_{m=1}^{M}P_{m}\leq NP^{\mathrm{ave}}, & \end{array}$$

where $\delta_{m}=\frac{{\left[{\mathrm{erfc}}^{-1}\left(2\epsilon \right)\right]}^{2}\sigma^{2}\eta }{g_{m}}$. Note that Problem P6 is still non-convex due to the coupling $P_{m}$ and $t_{m}^{a}$ in the constraint. However, an important property can be used to solve Problem P6, which is given in the following lemma, and for which the proof is omitted for simplification.

**Lemma** **3.** Problem P6 can be reformulated as a biconvex optimization problem.

For a biconvex optimization problem, multiple local maxima can be found—some of which may be the global optimum [27]. In order to find a potentially point solving Problem P6, Problem P6 is decomposed into two convex optimization problems with given part of the variables according to Lemma 3. First, given $\hat{P}$, Problem P6 is reduced to a convex problem as follows:

$$\begin{array}{cc} \left(P7\right)\;\;\max_{{\hat{t}}^{a}}\;\;\; & \sum_{m=1}^{M}t_{m}^{a}\left[\frac{1}{2}+\frac{1}{2}\mathrm{erf}\left(\sqrt{\frac{c_{m}P_{m}}{t_{m}^{a}}-b_{m}}\right)\right]\\ s.t.\; & \frac{\eta P_{m}h_{m}T}{P^{c}+\eta P_{m}h_{m}}\leq t_{m}^{a}\leq {\tilde{T}}_{m},\;\forall m, \end{array}$$

where ${\tilde{T}}_{m}=\min\left\{\frac{\eta P_{m}h_{m}T}{P^{c}+\delta_{m}},T\right\}$. Using the method for Problem P5, the solution for Problem P7 is given as: $t_{m}^{a}=\min\left\{\max\left\{\frac{\eta P_{m}h_{m}T}{P^{c}+\eta P_{m}h_{m}},\frac{c_{m}P_{m}}{{\tilde{z}}_{m}^{*}+b_{m}}\right\},{\tilde{T}}_{m}\right\}$, where ${\tilde{z}}_{m}^{*}$ is the solution for $f(z_{m},b_{m})=0$.

Given ${\hat{t}}^{a}$, Problem P6 is reduced to the other convex problem as follows

$$\begin{array}{ccc} \left(P8\right)\;\;\max_{\hat{P}}\;\;\; & \sum_{m=1}^{M}t_{m}^{a}\left[\frac{1}{2}+\frac{1}{2}\mathrm{erf}\left(\sqrt{\frac{c_{m}P_{m}}{t_{m}^{a}}-b_{m}}\right)\right] & \\ s.t.\; & P_{m}^{\mathrm{th}}\leq P_{m}\leq P^{\max}, & \forall m,\\ & P_{m}\geq \frac{\left(P^{c}+\delta_{m}\right)t_{m}^{a}}{\eta h_{m}T}, & \forall m,\\ & P_{m}\leq \frac{P^{c}t_{m}^{a}}{\eta h_{m}T-\eta h_{m}t_{m}^{a}}, & \forall m,\\ & \sum_{m=1}^{M}P_{m}\leq NP^{\mathrm{ave}}. & \end{array}$$

Using the method for Problem P2, the solution for Problem P8 can be obtained, and is given as: $P_{m}=\min\left\{\max\left\{P_{m}^{\min},\frac{\left({\tilde{\nu }}_{m}^{*}+b_{m}\right)t_{m}^{a}}{c_{m}}\right\},P_{m}^{\max}\right\}$, where $P_{m}^{\min}=\max\left\{P_{m}^{\mathrm{th}},\frac{\left(P^{c}+\delta_{m}\right)t_{m}^{a}}{\eta h_{m}T}\right\}$ and $P_{m}^{\max}=\min\left\{\frac{P^{c}t_{m}^{a}}{\eta h_{m}T-\eta h_{m}t_{m}^{a}},P^{\max}\right\}$, ${\tilde{\nu }}_{m}^{*}$ is the solution for equation $G\left(\nu_{m},c_{m}\right)-{\tilde{\lambda }}^{*}=0$ with respect to $\nu_{m}$, ${\tilde{\lambda }}^{*}$ is the optimal Lagrange multiplier.

In the state-of-the-art literature, the BCD method is extensively used to solve biconvex problems due to its superior performance. Therefore, an algorithm-based BCD is proposed to solve Problem P6, which is summarized in Algorithm 2. After deriving the optimal $P_{m}^{*}$ and $t_{m}^{a*}$, the optimal reflection ratio $n_{m}^{*}$ is given as $n_{m}^{*}=\frac{T}{t_{m}^{a*}}-\frac{P^{c}}{\eta P_{m}^{*}h_{m}}$. The asymptotic complexity of Algorithm 2 is analyzed as follows. The complexity of solving Problem P7 is O(ΘM), where Θ is the number of iterations for the bisection method used for Problem P7. The complexity of solving Problem P8 is O(ΔM), as analyzed for Algorithm 1. We assume that Ξ iterations is needed for Algorithm 2 to converge. The total complexity of Algorithm 2 is thus O((Δ+Θ)MΞ).

Combining Algorithms 1 and 2, Problem P3 can be solved, and the close-to-optimal solution is finally derived. Following the assumptions for Algorithm 1, the asymptotic complexity for solving Problem P3 is O((Δ+Θ)MΞK).

**Algorithm 2** Algorithm for Problem P6
1:Initialize: Set j=0, choose ${\hat{P}}^{\left(j\right)}=\left[P_{1}^{\left(j\right)},\ldots ,P_{M}^{\left(j\right)}\right]$, ${\hat{t}}^{a\left(j\right)}=\left[t_{1}^{a(j)},\ldots ,t_{M}^{a(j)}\right]$ satisfying the constraints of Problem P6, and compute $G^{\mathrm{sum}(j)}=\sum_{m=1}^{M}t_{m}^{a(j)}\left[\frac{1}{2}+\frac{1}{2}\mathrm{erf}\left(\sqrt{\frac{c_{m}P_{m}^{\left(j\right)}}{t_{m}^{a(j)}}-b_{m}}\right)\right]$, set the threshold ϵ>0 and $G^{\mathrm{sum}(j+1)}=G^{\mathrm{sum}(j)}+2\epsilon$.2:**while** $|G^{\mathrm{sum}(j+1)}-G^{\mathrm{sum}(j)}|>\epsilon$ **do**3: Keep ${\hat{P}}^{\left(j\right)}$ fixed, solve Problem P7 to derive the optimal time allocation ${\hat{t}}^{a\left(j+1\right)}$.4: Keep ${\hat{t}}^{a\left(j+1\right)}$ fixed, solve Problem P8 to derive the optimal power allocation ${\hat{P}}^{\left(j+1\right)}$.5: j=j+1.6: Update $G^{\mathrm{sum}(j+1)}$ with ${\hat{P}}^{\left(j\right)}$, ${\hat{t}}^{a\left(j\right)}$.7:**end** **while**8:**return** ${\hat{P}}^{*}={\hat{P}}^{\left(j\right)}$, ${\hat{t}}^{a*}={\hat{t}}^{a\left(j\right)}$ and $G^{\mathrm{sum}*}=G^{\mathrm{sum}(j+1)}$.


## 7. Simulation Results

In this section, simulation results are given to corroborate the superiority of the proposed resource allocation policies. The simulation environment is set as follows. For convenience, we assume that the duration of each tag is T=1 s. The forward and backward channel gains are set as $h_{i}=g_{i}=10^{-3}d_{i}^{-3}$, where $d_{i}$ is the distance between the reader and *i*-th tag. $d_{i}$ is randomly generated from 4 m to 6 m. The energy harvesting efficiency and maximum BER are set as η=0.5 and ϵ=0.3, respectively. Moreover, we set the circuit and noise power as $P^{c}=-20$ dBm and $\sigma^{2}=-90$ dBm, respectively. In addition, all of the experimental parameters are listed in Table 1. For performance comparison, the equal power allocation policy is considered. All of simulation curves are adopted 1000 Monte Carlo runs.

**Figure 3:**
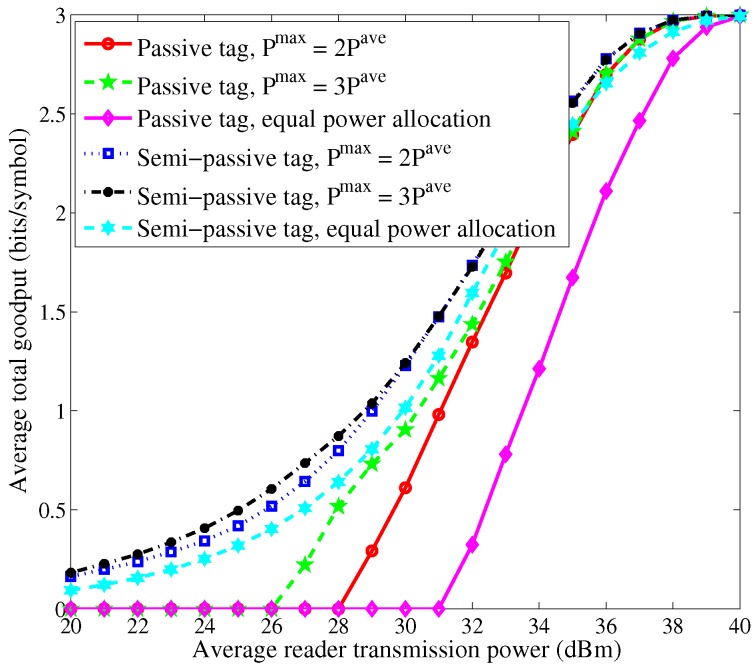
Average total goodput vs. average reader transmission power with N=3.

**Figure 4:**
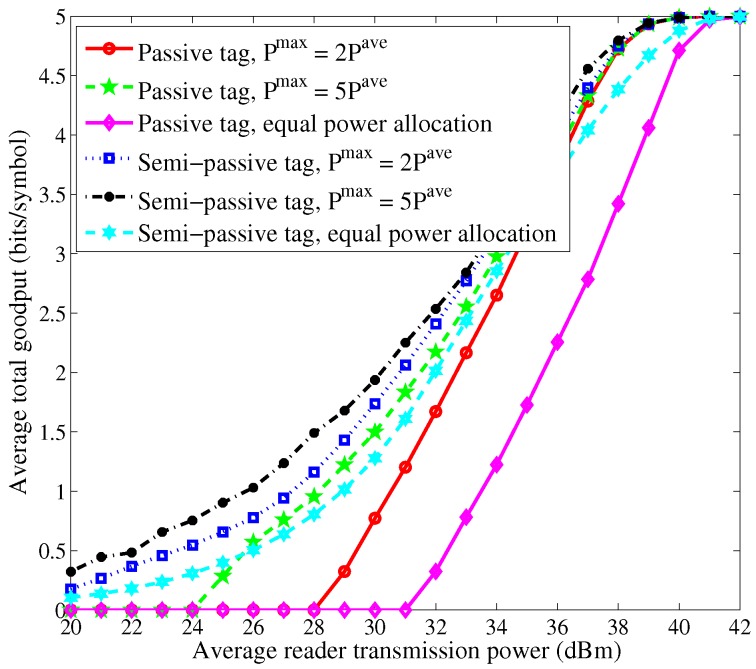
Average total goodput vs. average reader transmission power with N=5.

Figure 3 and Figure 4 show the curves of average total goodput versus the average reader transmission power. We can observe that as the average reader transmission power increases, the average total goodput first increases and then becomes invariant after the average reader transmission power exceeds some threshold. This is because the BER of each tag is close to 0 for high signal-to-noise ratio (SNR) when high allocated power is available. For low average reader transmission power, the passive tags cannot work. The performance of the case of semi-passive tags is superior to the case of passive tags due to the adoption of *sleep-then-active* strategy. Moreover, the proposed schemes yield much larger goodput than the scheme of equal power allocation.

## 8. Conclusions

This work studies resource allocation policies for multi-user BackCom systems by considering cases of both passive and semi-passive tags. To account for link reliability, the optimization problems are formulated to maximize the total system goodput of the two cases. For the passive tags case, we derive the optimal resource allocation policy by exploiting the priority of allocated reader transmission power. The “activate or not” decision is further discussed. For the semi-passive tags case, combing the priority of allocated reader transmission power and the proposed BCD-based optimization algorithm, we find the close-to-optimal solution. The simulation results confirm that the proposed policies can achieve larger total system goodput than conventional techniques.

## Figures and Tables

**Table 1:** Simulation parameters.

Parameters	Values
Time duration	T=1 s
Distance	4–6 m
Forward channel gains	$10^{-3}d_{i}^{-3}$
Backward channel gains	$10^{-3}d_{i}^{-3}$
Energy harvesting efficiency	η=0.5
Maximum BER	ϵ=0.3
Circuit power	$P^{c}=-20$ dBm
Noise power	$\sigma^{2}=-90$ dBm

## Appendix A

For the *i*-th sub-problem, if $NP^{\mathrm{ave}}\geq \left(N-i+1\right)P^{\max}$, the maximum total goodput is given as $\sum_{m=1}^{N-i+1}{\hat{G}}_{m}\left(P^{\max}\right)$. For the $\hat{i}$-th sub-problem, $\hat{i}=i+1,\cdots ,N$, $NP^{\mathrm{ave}}\geq \left(N-\hat{i}+1\right)P^{\max}$ is still satisfied, and the maximum total goodput is given as $\sum_{m=1}^{N-\hat{i}+1}{\hat{G}}_{m}\left(P^{\max}\right)$. It is obvious that $\sum_{m=1}^{N-i+1}{\hat{G}}_{m}\left(P^{\max}\right)>\sum_{m=1}^{N-i}{\hat{G}}_{m}\left(P^{\max}\right)>\cdots >{\hat{G}}_{1}\left(P^{\max}\right)$. Hence, we conclude that the solution for the $\hat{i}$-th sub-problem is not the optimal solution for Problem P1.

## Appendix B

The Lagrange function of Problem P2 is expressed as

$$L\left(\hat{P}\right)=\sum_{m=1}^{M}T\left[\frac{1}{2}+\frac{1}{2}\mathrm{erf}\left(\sqrt{a_{m}P_{m}-b_{m}}\right)\right]-\lambda \left(\sum_{m=1}^{M}P_{m}-NP^{\mathrm{ave}}\right)$$

where *λ* denotes the Lagrange multiplier. Problem P2 can be solved by exploiting its Karush–Kuhn–Tucker (KKT) conditions [26], which are given by

(B1) $$\begin{array}{c} \frac{\partial L}{\partial P_{m}^{*}}\left\{\begin{array}{c} <0,\;P_{m}^{*}=P_{m}^{\mathrm{th}},\\ =0,\;P_{m}^{\mathrm{th}}\leq P_{m}^{*}\leq P^{\max},\\ >0,\;P_{m}^{*}=P^{\max}, \end{array}\right.\;\forall m, \end{array}$$

(B2) $$\begin{array}{c} \lambda^{*}\left(\sum_{m=1}^{M}P_{m}^{*}-NP^{\mathrm{ave}}\right)=0, \end{array}$$

where $P_{m}^{*}$ and $\lambda^{*}$ denote the optimal primal and dual solutions of Problem P2, respectively. It is easy to verify that $\sum_{m=1}^{M}P_{m}^{*}=NP^{\mathrm{ave}}$ holds for Problem P2. Hence, we can assume $\lambda^{*}>0$ from Equation (B2) without loss of generality. According to Equation (B1), if $P_{m}^{\mathrm{th}}\leq P_{m}^{*}\leq P^{\max}$, it follows that

(B3) $$\begin{array}{c} G\left(a_{m}P_{m}^{*}-b_{m},c_{m}\right)=\lambda^{*},\;\forall m, \end{array}$$

where $G(\nu_{m},c_{m})$ with respect to $\nu_{m}$ is defined in Theorem 2. Define $\nu_{m}^{*}$ as the solution for the equation $G\left(\nu_{m},c_{m}\right)=\lambda^{*}$. Since $G(\nu_{m},c_{m})$ is a monotonically decreasing function of $\nu_{m}$, it is obtained that $\nu_{m}^{*}$ is the unique solution. Based on $\nu_{m}^{*}$, $P_{m}^{*}=\frac{\nu_{m}^{*}+b_{m}}{a_{m}}$. Combining with $P_{m}^{\mathrm{th}}\leq P_{m}^{*}\leq P^{\max}$, the optimal solution for Problem P2 is given as $P_{m}^{*}=\min\left\{\max\left\{P_{m}^{\mathrm{th}},\frac{\nu_{m}^{*}+b_{m}}{a_{m}}\right\},P^{\max}\right\}$.

## Appendix C

Assume the optimal solution for Problem P4 is $\left\{{\hat{P}}_{m},{\hat{t}}_{m}^{a},{\hat{n}}_{m}\right\}$ satisfying $P^{c}{\hat{t}}_{m}^{a}<\eta {\hat{P}}_{m}h_{m}\left(T-{\hat{t}}_{m}^{a}\right)+\eta \left(1-{\hat{n}}_{m}\right){\hat{P}}_{m}h_{m}{\hat{t}}_{m}^{a}$, ∀m. It is to prove that $\left\{{\hat{P}}_{m},{\hat{t}}_{m}^{a},{\hat{n}}_{m}\right\}$ is not the optimal solution by contradiction. It is not difficult to find $P_{m}^{*}$, $t_{m}^{a*}$, and $n_{m}^{*}$, satisfying that $t_{m}^{a*}=\frac{\eta P_{m}^{*}h_{m}T}{P^{c}+\eta P_{m}^{*}h_{m}n_{m}^{*}}$ with $n_{m}^{*}={\hat{n}}_{m}$, and $P_{m}^{*}={\hat{P}}_{m}$. Since $t_{m}^{a}\left[\frac{1}{2}+\frac{1}{2}\mathrm{erf}(\sqrt{a_{m}P_{m}n_{m}})\right]$ is an increasing function of $t_{m}^{a}$ and $n_{m}$, respectively, it is evident that $\sum_{m=1}^{M}t_{m}^{a*}\left[\frac{1}{2}+\frac{1}{2}\mathrm{erf}(\sqrt{a_{m}P_{m}^{*}n_{m}^{*}})\right]>\sum_{m=1}^{M}{\hat{t}}_{m}^{a}\left[\frac{1}{2}+\frac{1}{2}\mathrm{erf}(\sqrt{a_{m}{\hat{P}}_{m}{\hat{n}}_{m}})\right]$. Therefore, the assumption is contradiction, completing the proof.
